# Diverse functions of myeloid-derived suppressor cells in autoimmune diseases

**DOI:** 10.1007/s12026-023-09421-0

**Published:** 2023-09-21

**Authors:** Xin Xiong, Yang Zhang, Yu Wen

**Affiliations:** 1 Department of Pediatrics, Tongji Hospital, Tongji Medical College, Huazhong University of Science and Technology, Wuhan, China; 2 Department of Anesthesiology, Tongji Hospital, Tongji Medical College, Huazhong University of Science and Technology, Wuhan, China

**Keywords:** Myeloid-derived suppressor cell (MDSC), Suppressive, Pathogenic, Pro-inflammatory, Autoimmune disease

## Abstract

Since myeloid-derived suppressor cells (MDSCs) were found suppressing immune responses in cancer and other pathological conditions, subsequent researchers have pinned their hopes on the suppressive function against immune damage in autoimmune diseases. However, recent studies have found key distinctions of MDSC immune effects in cancer and autoimmunity. These include not only suppression and immune tolerance, but MDSCs also possess pro-inflammatory effects and exacerbate immune disorders during autoimmunity, while promoting T cell proliferation, inducing Th17 cell differentiation, releasing pro-inflammatory cytokines, and causing direct tissue damage. Additionally, MDSCs could interact with surrounding cells to directly cause tissue damage or repair, sometimes even as an inflammatory indicator in line with disease severity. These diverse manifestations could be partially attributed to the heterogeneity of MDSCs, but not all. The different disease types, disease states, and cytokine profiles alter the diverse phenotypes and functions of MDSCs, thus leading to the impairment or obversion of MDSC suppression. In this review, we summarize the functions of MDSCs in several autoimmune diseases and attempt to elucidate the mechanisms behind their actions.

## Introduction

Myeloid-derived suppressor cells (MDSCs), a heterogenic collection of immature myeloid cells with suppressive activity, have been found swiftly differentiating into mature granulocytes, macrophages, and dendritic cells (DCs) in normal physiological conditions, while expanding and accumulating in pathological conditions such as tumor, chronic inflammation, autoimmunity, transplantation, infection, trauma, and sepsis [1]. Only very few MDSCs are present in the steady state of healthy individuals and would be arrested and expanded at an immature phase of differentiation in pathologic conditions.

On the base of different morphologies and functions, MDSCs are roughly divided into two subsets, polymorphonuclear MDSCs (PMN-MDSCs) and monocytic MDSCs (M-MDSCs), along with different mechanisms in immune suppression. Correspondingly, these two subsets of MDSCs have their own characteristics of immunophenotype. In human, PMN-MDSCs are defined as CD11b^+^CD15^+^HLA-DR^low^CD66b^+^, and M-MDSCs as CD11b^+^CD14^+^CD33^+^HLA-DR^low/neg^. By contrast, the surface marker of MDSCs is Gr-1^+^CD11b^+^, which is simpler in mouse. PMN-MDSCs are described as CD11b^+^Ly6C^low^Ly6G^+^ and M-MDSCs as CD11b^+^Ly6C^hi^Ly6G^−^. Besides, increasing markers on MDSCs have been discovered closely relevant to immune suppression, such as IL4Rα, PD-L1, Lectin-type oxidized LDL receptor-1 (LOX-1), and inhibitor of differentiation1 (ID1). IL4Rα is only confined to M-MDSCs in tumor-bearing patients, but not PMN-MDSCs [2]. The increase of ID1 expression on CD33^+^CD11b^+^CD14^+^HLA-DR^low^ M-MDSCs is strongly associated with the upregulation of S100A8/9 and iNOS expression, which represents a more immunosuppressive potential in advanced melanoma [3]. For hepatocellular carcinoma patients, LOX-1^+^CD15^+^ PMN-MDSCs were positively related to overall survival as a result of inhibiting T cell proliferation through ROS/Arg I pathway induced by endoplasmic reticulum (ER) stress [4]. Hypoxia caused a prominent up-regulation of PD-L1 on splenic MDSCs, which could express higher levels of IL-6 and IL-10, and suppress T cell proliferation and function [5]. Nonetheless, the obvious heterogeneity of MDSCs contributed to the plasticity and instability of immune suppression.

The key feature of MDSCs is immune suppression. The suppression of immune responses relies chiefly on various mediators via cell–cell contact, including arginase 1 (Arg-1), inducible nitric oxide synthase (iNOS), reactive oxygen species (ROS), transforming growth factor (TGF)-β, IL-10, cyclo-oxygenase 2 (Cox2), and prostaglandin E2 (PGE2). PMN-MDSCs retain the immune function through the high release of ROS and Arg-1, while M-MDSCs via nitric oxide and Arg-1 production [1]. Moreover, MDSCs could also induce and cooperate with Tregs, via TGF-β and IL-10 [6]. To date, the role of MDSCs in cancer is primarily focused on the inhibition of effector T and B cells and induction of Tregs development in immune responses. However, the role of MDSCs in autoimmunity remains controversial. We explore current reports based on the functions of MDSCs in a range of autoimmune diseases.

## MDSCs in systemic lupus erythematosus (SLE)

Systemic lupus erythematosus (SLE) is a potentially fatal disease with variable clinical symptoms, characterized by hyper-activated immune responses and excessive production of pathogenic autoantibodies against self-antigens [7]. Several antigens, consisting of both nuclear and cytoplasmic antigens, could be presented to trigger host CD4^+^ T cells to expand autoantibody-inducing CD4^+^ T cells, leading to aberrant B cell responses with multiple autoantibodies generation and the final maturation of CD8^+^ T cells to cytotoxic T lymphocytes [8, 9]. In addition to immune suppression, MDSCs are more likely to provide a rebalance of the immune responses to maintain a durable remission and thus prevent tissue injury [10].

### The immune suppression of MDSCs in SLE

The observations regarding MDSC functions during the early years showed that M-MDSCs were positively correlated with disease severity in SLE patients and in a pristane-induced lupus mice model, acting as a protector with immunosuppressive function [11, 12]. Splenic CD11b^+^Gr-1^+^ MDSCs exhibit suppressive functions by expanding regulatory B cells (Bregs), reducing effector B cells (germinal center B cells and plasma cells), and suppressing effector T cells (follicular helper T cells, Th1 cells, and Th17 cells) in an iNOS-dependent manner [11, 13]. In cutaneous lupus erythematosus, DC-HIL as one of multiple co-receptors on M-MDSCs was upregulated to facilitate and ensure the inaction of T cell responses [14]. However, blocking the DC-HIL receptor could not recover the defect of T cell function at maximum. An extended study indicated that in two cases from three SLE patients, the increase of M-MDSCs and DC-HIL^+^ M-MDSCs did not give rise to IFN-γ expression and perform immunosuppressive properties, in M-MDSC-T cell proliferation suppression assays. Compared with expanded MDSCs in SLE, PD-L1+ MDSCs in control mice are more potent to suppress double-negative (CD4−CD8−CD3+) T cells and expand both Treg cells and regulatory B cells [15]. Meanwhile, the expression of suppression-related molecules (arginase-1, IDO, PD-L1, and IL-10) in MDSCs was found to be profoundly decreased in lupus patients and mice [15]. These data show that the functions of MDSCs are impaired during lupus progress, indicating that immune suppression is of a precarious characteristic in these cells.

Apart from adaptive immunity, MDSCs also interact with other innate immune cells and alter the inflammatory profiles during the innate immune responses. Under chronic exposure to interferon gamma (IFNγ), generated PMN-MDSCs also engage with macrophages to reprogram and polarize to peripheral alternatively activated macrophages which suppress the inflammation, promote tissue repair, remodeling, vasculogenesis, and retain homeostasis, via lessening CD40 expression and impairing IL-27 production, consequently facilitating immune evasion and causing dysfunctional myeloid responses in a SLE-prone model [16]. However, the study of MDSCs in innate immunity is still poor.

### The pro-inflammatory effect of MDSCs in SLE

Recently, it has been reported that MDSCs may exert pro-inflammatory functions during SLE progression. In pristane-induced lupus mice with C-type lectin receptor Dectin3 deficiency, the expansion of LOX-1^+^ M-MDSCs via silencing FoxO1 induced the differentiation of Th17 cells and exacerbated the severity of lupus [17]. In SLE patients, MDSCs produced higher arginase-1 (Arg-1) levels and increased the potential to promote Th17 differentiation via Arg-1 [18]. This was consistent with a study illustrating that MDSC-derived Arg-1 regulated miR-322-5p expression via the transforming growth factor (TGF)-β/SMAD signaling pathway, to manipulate Th17 cell and Treg differentiation, eventually worsening SLE disease [19]. IFN-γ could elevate the expression of reactive oxygen species (ROS) in splenic PMN-MDSCs by activating NADPH oxidative pathways, causing impaired differentiation of Treg [20]. Moreover, ROS secreted by MDSCs can directly induce podocyte injury by activating p-38MAPK and NF-kB signaling in lupus nephritis [21]. Other researchers also found that inhibition of Notch signaling could control the differentiation of MDSCs and decrease the production of ROS, to relieve lupus progress [22]. In brief, these studies suggested pathogenic roles of M-MDSCs and PMN-MDSCs in the regulation of lupus progress, especially PMN-MDSCs via ROS release. Instead of inducing T cell activation, PMN-MDSCs could also promote IFN-I signaling activation of B cells and contribute to disease progression through the lncRNA NEAT1-BAFF axis [23].

Taken together, the phenotype of MDSCs includes PD-L1, DC-HIL, Arg-1, and LOX-1, not limited exclusively to PMN-MDSCs and M-MDSCs. Different phenotypes of MDSC possess distinct potential of immune suppression, or even as a pro-inflammatory factor by expressing inflammatory cytokines (including IL-1β and ROS) and disrupting the adaptive immune responses in SLE progression (Table 1). The suppressive mechanisms include polarizing alternatively activated macrophages, inducing Treg and Breg cells, reducing effector B cells with autoantibody levels, inhibiting CD4^+^ T cell proliferation, and suppressing follicular helper T cells, Th1, and Th17 cells via the release of IFNγ, arginase-1, IDO, PD-L1, and IL-10. Meanwhile, MDSCs could display a stimulatory capacity of causing podocyte injury via ROS and promoting Th17 differentiation and B cell activation via IL‐1β, arginase-1, and IFN-I. There are two studies revealing the simultaneous and controversial effects of MDSCs in the same case of SLE, although this is due to the different phenotypes of PMN-MDSCs and M-MDSCs [20, 24]. Not only that, but there is another reason to explain this controversy, which is that SLE activity might account for the controversy of MDSC functions. Under some situations, MDSCs owing the same phenotype could exert such contrasting functions in different cases. However, the plasticity of MDSCs in SLE needs further experimental support.

**Table 1:** Role of MDSCs in SLE

Species	Organ	MDSCs	Effect	Mechanism	Ref
MRL/*lpr* mice and Roquin^san/san^ mice	Spleen, bone marrow (BM)	PD-L1^+^ MDSCs	Suppressive	Induce Treg cells and regulatory B cells; reduce autoantibody levels and degree of proteinuria; suppress double negative (CD4^−^CD8^−^CD3^+^) T cells	[15]
NZB × NZWF1 mice	Spleen	PMN-MDSCs	Suppressive	Inhibit cytokine-induced differentiation of naïve B cells into antibody-secreting cells in vitro	[25]
Pristane-induced lupus mice	Spleen, blood	M-MDSCs	Suppressive	Suppress T cell proliferation, inhibit Th1 differentiation but enhance Treg development	[12]
Roquin^san/san^ mice	BM	MDSCs	Suppressive	Expand IL-10 producing B cells; decrease effector B cells such as germinal center B cells and plasma cells; decrease follicular helper T cells, Th1, and Th17 cells	[13]
SLE patients	Blood	M-MDSCs	Suppressive	Inhibit CD4^+^ T cell proliferation	[11]
SLE patients	Blood, skin	DC-HIL^+^ M-MDSCs	Suppressive	Suppress T cells	[14]
*ifna*^*r*−/−^ARE mice	BM	PMN-MDSCs	Suppressive	Reprogram SLE macrophage polarization via CD40/IL-27 axis to establish immune evasion	[16]
Pristane-induced lupus mice	Spleen, BM	MDSCs	Pro-inflammatory	Induce podocyte injury via ROS	[26]
Pristane-induced lupus mice	Spleen	Arg-1^+^ MDSCs	Pro-inflammatory	Manipulate Th17 cells, Tregs, and the Th17/Treg ratio via TGF-β/SMAD pathway	[19]
Imiquimod/pristane-induced lupus mice	Spleen, kidney	MDSCs	Pro-inflammatory	ROS promotes podocyte injury via p-38MaPK and NF-kB signaling	[21]
MRL/*lpr* mice	Spleen	PMN-MDSCs	Pro-inflammatory	Promote IFN-I signaling activation of B cells	[23]
SLE patients	Blood	MDSCs	Pro-inflammatory	Promote Th17 differentiation via Arg-1 in vitro	[18]
Pristane-induced lupus mice	Spleen, kidney, BM	LOX-1^+^ M-MDSCs	Pro-inflammatory	Promote Th17 differentiation	[17]
NZB/W F1 lupus-prone mice	Spleen, BM	MDSCs	Contradictory	PMN-MDSCs: promote the expansion and proliferation of CD4^+^ T cells in vitro; M-MDSCs: slightly suppress CD4^+^ T cell proliferation	[24]
SLE patients, MRL/*lpr* mice	Blood	PMN-MDSCs, M-MDSCs	Contradictory	PMN-MDSCs: suppress T cell activation in vivo, impair Treg differentiation via ROS in vitro; M-MDSCs: polarize Th17 cells by IL‐1β in vitro	[20]

## MDSCs in rheumatoid arthritis (RA)

Rheumatoid arthritis (RA) is a chronic inflammatory arthropathy, and abundant evidence has demonstrated that immune disorders involve pro-inflammatory roles in the pathogenesis of arthritis, including T cells, B cells, macrophages, osteoclasts (OCs), dendritic cells (DCs), and other cell types [27, 28]. In essence, effector T cells, together with B cells and other innate effector cells, trigger the activation of resident fibroblast-like synoviocytes; thus, spontaneous chronic inflammation persists within the synovial membrane [29]. MDSCs are responsible for regulating immune responses and employ several means to suppress antigen-dependent and antigen-independent T cell activation, as a method for cellular therapy to treat RA.

### The suppressive effect of MDSCs in arthritis

Initial evidence for the involvement of MDSCs in arthritis came from a study first described by Katalin Mikecz [30] in 2012. In proteoglycan-induced arthritis (PGIA) mice, the excised synovial fluid (SF) contained a population of PMN-MDSCs that potently suppressed DC maturation and Ag- and DC-dependent T cell proliferation via iNOS and ROS [30]. MDSCs have also been found to accumulate in the spleens of mice with collagen-induced arthritis (CIA), where they inhibit CD4^+^ T cell pro-inflammatory immune responses, including promotion of IL-10 production by the CD4^+^ T cells and suppression of Th17 cell differentiation [31]. Even in synovial fluid (SF) of RA patients, MDSCs are capable of limiting the expansion of joint-infiltrating T cells which are most likely pathogenic [32]. Furthermore, in vivo infusion of MDSCs resulted in decreased Th1 and Th17 cell numbers but increased Tregs via IL-10, thus markedly ameliorating inflammatory arthritis [33]. It has also been demonstrated that M-MDSCs suppressed autologous B cell proliferation and antibody production, which was dependent on nitrous oxide (NO), prostaglandin E2 (PGE2), and cell–cell contact [34]. However, PMN-MDSC-derived exosomes (exo) have been shown to inhibit Th1 and Th17 cell responses through miR-29a-3p and miR-93-5p by targeting T-bet and STAT3, and upregulating GSK-3β and CREB phosphorylation levels to generate IL-10^+^ Bregs via production of exosome PGE2 [35, 36]. Hence, MDSC can modulate innate and adaptive immune responses via various cytokines, to protect against disease progression.

### The indicative, pro-inflammatory, and osteoclastic effects of MDSCs in arthritis

Other than MDSC immunosuppression from above-mentioned studies, several reports revealed the disparate effects of MDSC in arthritis. Early, Eishi Ashiharathe [31] demonstrated that depletion of MDSCs at the initial disease stage could abrogate the spontaneous improvement of CIA, which broke the rules in terms of its suppressive effect. In mice and human patients with RA, MDSCs correlated positively with regard to disease severity and inflammatory Th17 response, the latter of which have the capacity to produce inflammatory cytokines (IL-1β, TNF-α) and therefore driving Th17 cell differentiation [37]. Besides, IL-1β derived by MDSCs likely accounts for the induction of Th17 differentiation [38]. Moreover, the functions of MDSCs are not restricted to the regulation of immune responses. MDSC-Th17 interaction stimulated the pro-osteoclastogenic signal RANK-L on Th17 cells, which in turn reprogramed MDSCs into osteoclasts with bone-resorbing activity via NF-κB and IL-1 signal pathways [39, 40]. From a bivariate analysis of clinical characteristics in RA patients, the proportions of MDSCs and M-MDSCs in RA patients were directly related to the patients’ joint inflammation indexes and disease activity, as an indicator for accessing arthritis [41].

Therefore, these data reveal that MDSCs exert indicative, pro-inflammatory, and osteoclastic effects in arthritis, not limited to immune suppression (Table 2). Aside from suppressing T cell proliferation and reducing Th1 and Th17 cell differentiation via IL-10, NO, and IFN-γ, MDSCs could inhibit autologous B cell proliferation and antibody production via NO and PGE2, while PMN-MDSCs promote IL-10^+^ Breg cell differentiation via exosome PGE2. The pro-inflammatory effects of MDSCs involve secreting inflammatory cytokines (IL-1β, TNF-α) at high levels, inducing Th17 differentiation, and interacting with Th17 cells to activate the pro-osteoclastogenic signal, or even directly polarizing into osteoclasts with a bone-resorbing potential. However, a previous study reported that both MDSC depletion via anti-Gr-1 Abs and adoptive transfer of MDSCs could hinder the arthritis progress in the same CIA model [31]. Several factors can be responsible for this contradiction in CIA, including the unreliability of MDSC phenotypes, the conversion of MDSC development, the diversity of MDSC function, and the stage of disease progress. The depletion of MDSCs was administrated from day 35 after the first immunization, while adoptive MDSCs were transferred on day 0 and day 21 [31]. It might be possible for MDSCs keeping suppressive at early stage of CIA progress, but acquiring a pro-inflammatory capacity at later stage of CIA progress. The difference of these contradictory results is up to the various microenvironmental conditions. Further studies are needed to further elucidate the phenotypes and roles of MDSCs in arthritis.

**Table 2:** Role of MDSCs in arthritis

Species	Organ	MDSCs	Effect	Mechanism	Ref
Zymosan-induced ILD in SKG mice	Lung	MDSCs	Suppressive	Suppress T cell proliferation and Th17 cell differentiation in vitro	[42]
CIA	Spleen	MDSCs	Suppressive	Reciprocally regulate Th17/Treg cells and T cell proliferation via IL-10	[33]
CIA	Spleen	PMN-MDSCs	Suppressive	Exosomal PGE2 promote the generation of IL-10^+^ Breg cells	[36]
CIA and antigen-induced arthritis	Spleen	MDSCs	Suppressive	Decrease Th17 cell numbers and macrophages in the draining lymph nodes and joint tissue	[43]
CIA	BM	M-MDSCs	Suppressive	Suppress T cell proliferation via NO and IFN-γ, inhibit autologous B cell proliferation and antibody production via NO and PGE2	[34]
CIA	Spleen	PMN-MDSCs	Suppressive	Suppress polyclonal T cell proliferation, and suppress Th1 and Th17 cell differentiation	[35, 44]
Proteoglycan-induced arthritis	BM	MDSCs	Suppressive	Reduce PG-specific T cell responses and inhibit both antigen-specific and polyclonal T cell proliferation primarily via NO	[45]
RA patients	SF	MDSCs	Suppressive	Suppress the proliferation of alloantigen-induced autologous T cells	[32]
CIA	Spleen, BM	MDSCs	Pro-inflammatory	Produce high levels of inflammatory cytokines (e.g., IL-1β, TNF-α) and induce Th17 differentiation via IL-1β	[37, 38, 46]
RA patients, CIA mice	Blood	M-MDSCs	Pro-inflammatory	Differentiate into osteoclasts with bone resorbing activity, and MDSC-Th17 interaction upregulates the pro-osteoclastogenic signal RANK-L on Th17 cells	[39]
CIA	Spleen	MDSCs	Contradictory	Inhibiting CD4^+^ T cell response (proliferation, differentiation, reduced the production of IL-10, IFN-γ, IL-2, TNF-α, and IL-6), while MDSC depletion abrogate CIA improvement	[31]
RA patients	Blood	MDSCs, M-MDSCs	Indicative	Related to the patient’s joint inflammation indexes and disease activity	[41]
CIA	BM	MDSCs	Osteoclastic	Differentiate into TRAP^+^ osteoclasts and have bone resorption function	[40]

## MDSCs in multiple sclerosis (MS)

Our knowledge of multiple sclerosis (MS) highlights that it is a cell-mediated autoimmune disease accompanied by chronic inflammation, demyelination, axonal loss, and gliosis. Inflammation is generally considered as the main trigger leading to central nervous system (CNS) tissue damage, which is caused by the infiltration of cells, including Ag-specific and nonspecific CD4^+^ and CD8^+^ T cells, B cells, and antigen ­presenting cells [47, 48]. Except these cells, pro­inflammatory cytokines (such as IFN-γ, TNFα, IL-17, IL-21, IL-22, IL-6, GM-CSF), and cytolytic granules) could break down the blood–brain barrier (BBB), inducing further inflammation and demyelination, thus contributing toward CNS injury. In order to study the key pathological features of MS, experimental autoimmune encephalomyelitis (EAE) is usually used as a model to explore multiple facets of the immune and neural mechanisms in MS [49].

### The indicative and suppressive effects of MDSCs in MS/EAE

Since the first description of spinal cord-isolated MDSCs promoting T lymphocyte apoptosis in 2011, the function of MDSCs in MS has become a hot topic of research over the years. In terms of the quantity, MDSC accumulation in the spleen is directly indicative of the disease severity and outcome in EAE, with relation to lymphocyte infiltration, demyelination, and axonal damage within the CNS [50]. In particular, M‑MDSCs at baseline in MS patients are positive related with the therapeutic response to 12 months of fingolimod treatment [51]. This is a hint to us that M-MDSCs are intimately involved in the progression, therapy, and prognosis of MS. Functionally, splenic PMN-MDSCs were able to suppress antigen-specific Th1 and Th17 immune responses in EAE mice, which was reliant on upregulation of the programmed death 1 ligand (PD-L1) [52]. Moreover, Arg-I^+^ MDSCs in the spinal cord exhibited the distinctive MDSC surface markers Arg-I/CD11b/Gr-1/M-CSF1R, and were related to the EAE time course together with the proportion of apoptotic T cells [53]. It was not only T lymphocytes that were affected; MDSCs were also able to selectively control B cell accumulation within the CNS in EAE mice. After stimulation with GM-CSF and IL-6 from B cells, the recruited Ly6G^+^ cells obtained the properties of PMN-MDSCs in a manner dependent on the signal transducer STAT3, which in turn retained CD138^+^ B cell activation in the cerebrospinal fluid (CSF) [54].

As regards the suppressive activity of MDSCs, antigen-expressing MDSCs (IiMOG-MDSCs) from BM could possess a higher expression of PD-L1, CD80, CD86, and the MHC class II molecule I-A^b^, along with a higher pro-apoptotic effect on CD4^+^ T cells, which suggested that MDSC suppressive activity was not invariable [55]. Furthermore, a single injection of IFN-β at the onset of the clinical course increased the presence of MDSCs within the smaller demyelinated areas, thus reducing the severity of the EAE [56]. Hence, it follows that the function of MDSCs is diverse and dependent partly on the microenvironment.

### The pro-inflammatory and regenerative effects of MDSCs in MS/EAE

Under Th17-polarizing conditions, MDSCs induced Th17 differentiation from naive CD4^+^ T cell precursors through IL-1β, with the elevation of IL-17A production, thus contributing to the pathogenesis observed in EAE [57]. In the spinal cord of EAE mice, pseudolycorine chloride inhibited the expansion of MDSCs, thus suppressing Th17 cell differentiation and IL-17A secretion [58]. In line with the findings from this study, lung-derived PMN-MDSCs in EAE mice expanded and produced IL-6, promoting activated CD4^+^ T cell polarization toward Th17 cells and enhancing IL-17A production in the presence of TGF-β [59]. These observations showed that MDSCs derived from different tissues could collaborate with phenotype-altering cytokines to induce Th17 differentiation, with pro-inflammatory and pathogenic effects contributing to disease processes.

Except for immune function, a study on oligodendrocyte precursor cells (OPCs) and remyelination was conducted to investigate the role of MDSCs in myelin preservation and repair [60]. This study indicated that osteopontin secreted from MDSCs promoted OPC survival, proliferation, and differentiation. It is improper to generalize about the immune function of MDSCs in autoimmunity, whereby the disease state and cytokine profiles can all become influencing factors. The summary of MDSC function in MS patients and EAE model is presented in Table 3.

**Table 3:** Role of MDSCs in MS/EAE

Species	Organ	MDSCs	Effect	Mechanism	Ref
EAE, MS patients	Blood	M-MDSCs	Indicative	Represent a good therapeutic response to fingolimod	[51]
EAE	Spleen	MDSCs	Indicative, suppressive	Related to less myelin destruction and axonal damage; Induce T cell apoptosis	[50]
EAE	BM	IiMOG-MDSCs	Suppressive	Reduce the proportion of activated T cells and increases B cells with a regulatory phenotype	[55]
EAE	Spleen	PMN-MDSCs	Suppressive	Inhibit CD4^+^ T cell proliferation via Arg-1	[64]
EAE	CNS	PMN-MDSCs	Suppressive	Control the accumulation and cytokine secretion of CD138^+^ B cells	[54]
EAE	BM	miR-223^−/−^ M-MDSCs	Suppressive	Have more potent suppressive activity with increased *Arg1* and *Stat3* expression	[65]
EAE	Spleen	MDSCs	Suppressive	Augment T cell apoptosis	[56]
EAE	Spleen	DC-HIL^+^ MDSCs	Suppressive	Mediate the T cell suppressor function with upregulation of INF-γ, NO, and ROS expression	[66]
EAE	Spleen	PMN-MDSCs	Suppressive	Inhibit encephalitogenic Th1 and Th17 immune responses	[52]
EAE	Peritoneal, Spleen	MDSCs	Suppressive	Suppress CD4^+^ T cell proliferation via Arg-1and mediate Treg expansion	[67–69]
EAE	Spinal cord	Arg-I^+^ MDSCs	Suppressive	Promote T lymphocyte apoptosis	[53]
EAE	Spleen	M-MDSCs	Suppressive	Suppress T cell proliferation and induce T cell apoptosis via NO	[70]
EAE	Spleen, spinal cord	MDSCs	Pro-inflammatory	Facilitate Th17 differentiation	[57, 58]
EAE	Lung	PMN-MDSCs	Pro-inflammatory	Produce inflammatory cytokines and Th17 polarization	[59]
MS patients	Blood	M-MDSCs	Contradictory	SPMS: promoted autologous T cell proliferation; RRMS and HCs: T cell regulatory function	[61]
EAE	Spleen	MDSCs	Regenerative	Promote oligodendrocyte precursor cell (OPC) proliferation and differentiation	[60]

In brief, MDSCs have suppressive, pro-inflammatory, and regenerative functions in MS and EAE animal models. The suppressive effect is mainly supported by the next performances: MDSCs can inhibit CD4^+^ T cell proliferation, augment T cell apoptosis, expand Tregs, and inhibit encephalitogenic Th1 and Th17 immune responses. IiMOG-MDSCs can also facilitate B cells with a regulatory phenotype, while PMN-MDSCs restrain the accumulation and cytokine secretion of CD138^+^ B cells. The pro-inflammatory function is mainly confirmed by MDSCs which produce inflammatory cytokines and promote Th17 polarization.

Nevertheless, the effect of MDSCs is not always consistent, even in the same study. M-MDSCs in secondary progressive MS patients improved autologous T cell proliferation with downregulation of IL-10 and heme oxygenase 1 expression, in contrast with T cell suppression in relapsing remitting MS patients and healthy controls [61]. There are two similar examples about mononuclear phagocytes and myeloid cells help illustrate this controversy. Mononuclear phagocytes chiefly show an M^iNOS^ polarization in the spinal cord parenchyma at the initial stages of lesion formation, while often shift to an M^Arginase^ phenotype in the meninges during lesion resolution [62]. During EAE, CNS-infiltrating myeloid cells on the single cell level with a pro-inflammatory polarization shifted from iNOS to Arg1/CD206 expression with suppressive or pro-regenerative properties immediately prior to clinical remissions [63]. Hence, the phenotype of myeloid cells goes together with the local microenvironment: the local microenvironment influences the heterogeneity of myeloid cells, which in turn passively influent the development and resolution of inflammation in CNS.

## MDSCs in inflammatory bowel disease (IBD)

Inflammatory bowel disease (IBD) refers to a chronic inflammatory condition of the gastrointestinal tract, including ulcerative colitis (UC) and Crohn’s disease (CD). Although the pathogenesis is still obscure, IBD is a multifactorial, immune-mediated disease caused by gene susceptibility and environmental factors. At the early stage of IBD, impairment of intestinal barrier function leads to the translocation of commensal microorganisms, as the initial trigger activating innate and then adaptive immunity subsequently [71]. In particular, innate lymphoid cells, T cells, macrophages, neutrophils, and DCs may contribute to intestinal tissue destruction, while Th17 cells are implicated as playing priming and pathogenic roles in the gut [72]. Therefore, the regulation of activated immune cells is relevant for the suppression of intestinal inflammation in IBD.

### The suppressive effect of MDSCs in IBD

In colitis mice, the percentage of MDSCs was positively correlated with colitis severity, and adoptive transfer of MDSCs could protect from TNBS-induced intestinal inflammation via the downregulation of IFN-γ, IL-17, and TNF-α [73]. Repetitive transfer of CD8^+^ T cells could also induce an increase of suppressive activity in MDSCs [74]. And it could create an immune feedback loop that would alleviate intestinal inflammation further. Concerning MDSC subsets, different reports have drawn different manifestations. Among MDSCs, only Ly6C^high^ M-MDSCs suppress Th1 cell responses and promote Treg expansion to avoid excessive T cell activation via upregulation of iNOS and Arg-1 [75]. However, PMN-MDSC exosomes could transport Arg-1 and facilitate the spontaneous improvement of colitis via a similar immunoregulatory pathway [76]. Arg-1^+^ MDSCs have even modulated Th17 cell polarization to enhance IL-17A secretion, thereby attenuating the immune response and alleviating colitis [77]. In addition, acetylcholine and MDSCs were able to establish a neuroimmune regulatory pathway via increasing IL-10 release from M-MDSCs to alleviate colitis inflammation [78]. Collectively, these data indicate that modulation of MDSC suppression is an important option protecting against IBD.

### The pro-inflammatory and barrier-pathogenic effects of MDSCs in IBD

With the advances in IBD research, the role of MDSCs in colitis has become paradoxical. Firstly, the immunosuppression of MDSCs is not always available. Under a steady state, BM-MDSCs suppress activation and proliferation of CD4^+^T cells in a dose-dependent manner, while adoptive transfer of MDSCs into ongoing colitis mice aggravated the colitic phenotype, in consistence with CD33^+^CD15^+^ MDSCs from IBD patients enhancing T cell proliferation in vitro [79]. As a critical regulator of MDSC suppressive function, the lack of CEBPβ under an inflammatory milieu explains the reason of suppression abrogation observed in MDSCs. Likewise, a previous report showed that Ly6C^hi^ M-MDSCs during colitis extensively invaded the colon and switched from regulatory macrophages (MPs) to pro-inflammatory CD103^−^CX3CR1^int^CD11b^+^ DCs, producing high levels of IL-12, IL-23, iNOS, and TNF [80]. Collectively, this series of studies implied that the colonic milieu controlled the functional characteristics of MDSCs, including pro- or anti-inflammatory properties. Consistent with the evident MDSC pro-inflammatory function, hydrogen sulfide was able to locally limit the recruitment of PMN-MDSCs in the colon of *Helicobacter hepaticus* (*Hh*)-infected mice to retard disease progression, despite unknown reasons [81].

Secondly, MDSCs also exerted non-immune functions in colitis models. In *Il10*^*−/−*^* Il17a*^*−/−*^ mice, the high concentration of MDSC-expressed NO induced the barrier disruption of gut microbiota and exacerbated the pathology of the colitis [82].

Thus, the role of MDSCs is complex and might be closely associated with the local milieu and the specificity of IBD disease (Table 4). Most of the previous studies are based on the behavior of MDSC suppression, including attenuating T cell proliferation, preventing Th1 cell development, inducing Treg expansion, and producing iNOS, TGF-β, and IL-10, NO and ARG1 to interfere CD4^+^ and CD8^+^ T cell-mediated enterocolitis. The pathogenic evidence of MDSCs is scattered and superficial delineations, while the related study of pathogenic mechanism is still lack.

**Table 4:** Role of MDSCs in IBD

Species	Organ	MDSCs	Effect	Mechanism	Ref
DSS-induced colitis	BM	M-MDSCs	Suppressive	Enlarge IL-10 production	[78]
DSS-induced colitis	BM	PMN-MDSCs	Suppressive	Inhibit T cell responses by NO production	[83]
TNBS- and DSS-induced colitis	Spleen	MDSCs	Suppressive	Suppress the proliferation of lymphocytes	[73]
DSS-induced colitis	Spleen	PMN-MDSCs	Suppressive	Suppress delayed-type hypersensitivity, inhibit Th1 cell proliferation and promoting Tregs expansion	[76]
DSS-induced colitis	BM	MDSCs	Suppressive	Prevent Th1 cell development, promote Treg expansion, and produce iNOS, TGF-β, and IL-10 and ARG1	[84, 85]
*IL-10*^*−/−*^ mice	Spleen, colon	MDSCs	Suppressive	Attenuate T cell proliferation and reduce IFN-γ and GM-CSF production by LP-derived T cells	[86]
DSS-induced colitis	Spleen	MDSCs	Suppressive	Release Arg-1 to promote IL-17A accumulation and reduce IL-17F expression	[77]
VILLIN-HA mice	Spleen	MDSCs	Suppressive	Inhibit antigen-specific CD8^+^ T cell-mediated enterocolitis	[74]
T cell transfer colitis	Spleen, colon	M-MDSCs	Suppressive	Upregulate of iNOS and arginase-1, inhibit Th1 responses but enhance generation of Treg cells	[75]
*Il10*^*−/−*^* Il17a*^*−/−*^mice	Spleen, BM	MDSCs	Barrier-pathogenic	Express NO to disrupt the composition of gut microbiota	[82]
IBD patients	Blood	CD33^+^CD15^+^ MDSCs	Pro-inflammatory	Enhance T cell proliferation in vitro	[79]
*Hh*-infected mice	Colon	PMN-MDSCs	Pro-inflammatory	Unknown	[81]

## MDSCs in type 1 diabetes (T1D)

Type 1 diabetes (T1D) is an autoimmune disease exhibiting insulin resistance and hyperglycemia caused by T cell-mediated attack on islet β cells. The pathogenesis of type 1 diabetes results from a complex interplay between genome, metabolism, immune responses, and environmental factors [87]. The interactions between genes and environmental factors activate antigen-presenting cells to take up β cell peptides and react with autoreactive CD4^+^ T lymphocytes. This in turn leads to the activation of autoreactive CD8^+^ T cells, which are the crucial immune cells attacking islet β cells. Certainly, pro-inflammatory cytokines and ROS released from innate immune cells can then exacerbate β cell failure and destruction. Hence, MDSCs as immunosuppressive cells hold great potential for the treatment of insulin inflammation.

### The suppressive, preventive, and renal-protecting effects of MDSCs in T1D

The function of MDSCs in type1diabetes is generally immunosuppressive. It was reported that CD33^+^HLA-DR^−^ MDSCs were increased in the peripheral blood of type 1 diabetes affected patients with a predominance of the CD14^+^ M-MDSC subset [88]. A requirement of MDSCs suppressing T cell expansion is cell–cell contact but independent of ROS or NO [89]. In particular, the suppression of M-MDSCs to autologous T cells did not take effect until it is at the high MDSC:T cell ratio, whereas M-MDSC in T1D patients is less potent than M-MDSC from lung cancer [90]. Probably MDSCs under the cytokine profile of tumors could acquire stronger potential of immune suppression, compared with autoimmune diseases [91]. Another study conflicted this requirement about cell–cell contact, showing that MDSCs could suppress diabetogenic T cell response in an Arg/iNOS-dependent manner [92]. Anyway, the protective effect is accomplished by means of immune suppression on autoreactive T cells which attack islet β cells.

In NOD mice as a model of autoimmune diabetes, adoptive transfer of MDSCs prevented on onset of diabetes in 60% of the mice by mediating the development of Tregs and T cell anergy at lower MDSCs doses [93]. In line with the low efficacy displayed by MDSCs, suppressive function may be enhanced with cytokine induction, such as TGF-β [89, 92].

Apart from preventing the development of diabetes, cytokine-induced MDSCs could also mediate the glomerular filtration rate to degrade the kidney-to-body weight ratio and reduce the production of fibronectin in the renal glomerulus, preventing renal fibrosis of STZ-induced mice [94]. Particularly, the percentage of MDSCs in diabetic patients with nephropathy was positively correlated with levels of microalbumin [88]. However, the molecular mechanisms of renal protection remain unknown.

### The pro-inflammatory effect of MDSCs in T1D

In a hyperglycemic state, the inhibition of MDSCs on T cell response is attenuated and MDSCs release more IFN-γ to activate allogeneic T cells, contributing to an inflammatory environment [94]. It is worth mentioning that a high-glucose environment can induce M-MDSCs to differentiate into pro-inflammatory macrophages via the mTOR signaling pathway, causing difficulties during wound healing [95].

The contradiction of MDSCs has mostly focused on the immune function and renal protection. The cytokine profiles and glucose levels characterize the nature of MDSCs, including the effect on T cell proliferation and activation, and development, even self-polarization into pro-inflammatory macrophages, whereas the mechanism of renal protection is unknown. The summary of MDSC function in diabetes is tabulated in Table 5.

**Table 5:** Role of MDSCs in diabetes

Species	Organ	MDSCs	Effect	Mechanism	Ref
NOD mice	Spleen	MDSCs	Suppressive	Suppress T cell proliferation	[96]
NOD/SCID mice, T1D patients	Blood	FibrocyticMDSCs	Suppressive	Induce normoglycemia, promote Treg cell expansion and block CD8^+^ T cell proliferation via IDO	[97]
STZ-induced diabetes, T1Dpatients	Spleen, blood	MDSCs	Suppressive	Suppress T cell proliferation, especially CD8^+^ cell	[89, 98]
CD4-HA-TCR T cell transfer, NOD/SCID mice	Spleen and BM from colon cancer mice	MDSCs	Suppressive	Induce anergy in autoreactive T cells and the development of Tregs, inhibit lymphocyte infiltration and insulitis	[93]
STZ-treated C3^*−/−*^ mice	Spleen	MDSCs	Suppressive	Suppress diabetogenic T cell proliferation	[92]
T1D patients	Blood	M-MDSCs	Suppressive	Suppress CD4^+^ and CD8^+^ T cell proliferation and T cell pro-inflammatory cytokine production	[90]
STZ-induced diabetes	BM	M-MDSCs	Pro-inflammatory	Differentiate into pro-inflammatory macrophages under high glucose	[95]
STZ-induced diabetes	BM	MDSCs	Contradictory	Ameliorate renal fibronectin expression and have a reduced suppressive activity; induced more allogeneic T cell activation and create an inflammatory state	[94]

## Discussion

**Fig. 1:**
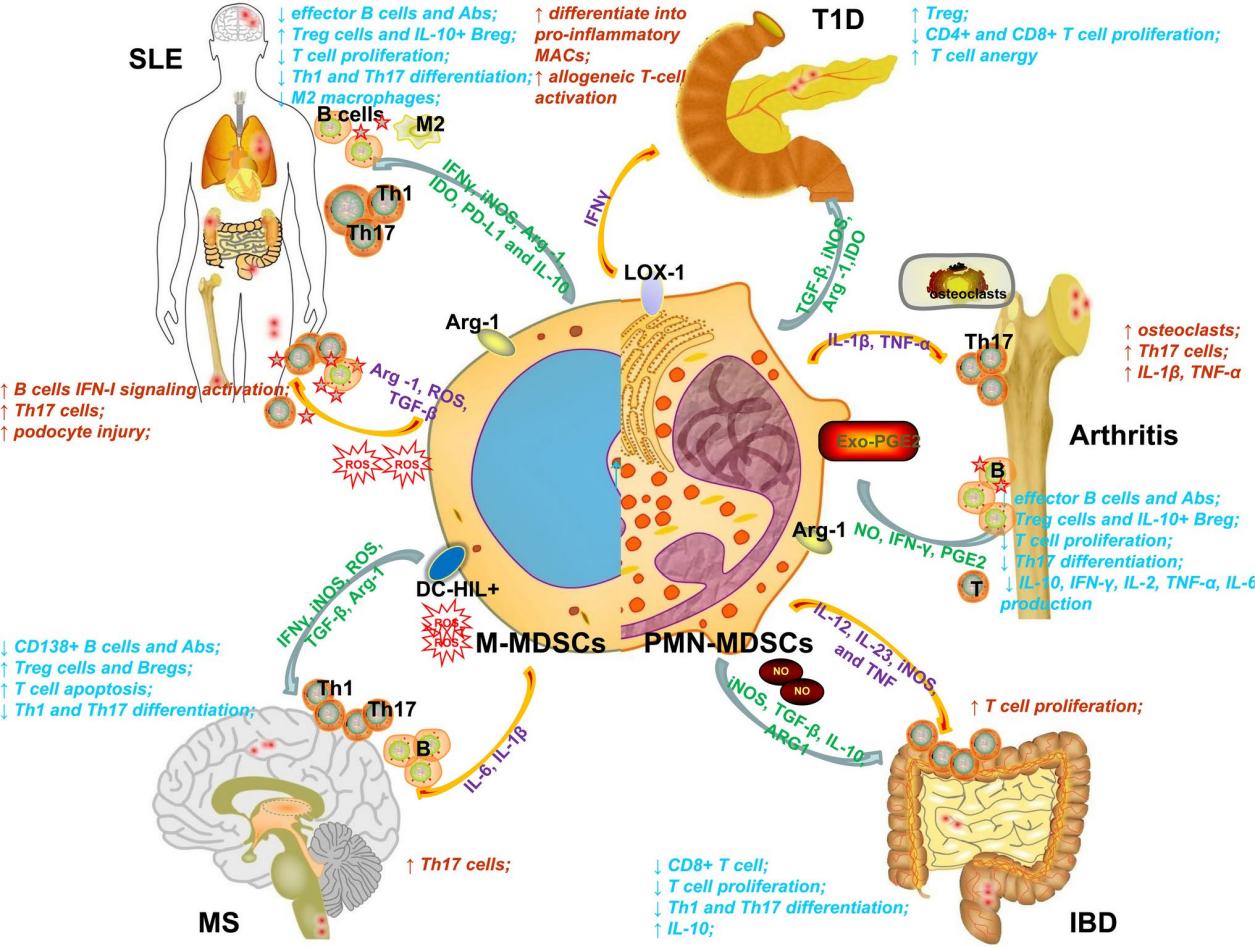
Functions of MDSCs in autoimmune diseases. MDSCs exert pro-inflammatory and suppressive effects via releasing cytokine, regulating T cells and B cell responses or directly injuring tissues. The blue arrows represent the immune suppression of MDSCs, while the yellow arrow represents the pro-inflammatory effects from MDSCs

Autoimmunity is a type of immune response that immune tolerance breaks down, and the resulting auto-reacting B or T cells can cause tissue damage. Reports into tumor biology have shown that MDSCs perform suppressive effects via various mechanisms, leading to the tolerance of the immune system and tumor cell invasion. Due to their immunosuppressive capacity, MDSCs are considered to be an appropriate cell population for restraining or delaying excessive damage. Nevertheless, the summary of autoimmune diseases has revealed the diversity and paradox involved in MDSC function (Fig. 1).

MDSCs are considered as a heterogeneous subset of cells involving myeloid progenitor cells and immature myeloid cells. The varying features of MDSCs are related to their various phenotypes. The most typical example is the differences in phenotypes and functions observed between PMN-MDSCs and M-MDSCs. PMN-MDSCs possess stronger potential for inhibiting polyclonal T cell proliferation in vitro, compared with M-MDSCs expressing higher surface levels of CD40 and CD86 [44]. Meanwhile, PMN-MDSCs expressed higher functional molecules and chemokine receptors including IL-10, TGF-β1, CCR5, and CXCR2 than those seen in M-MDSCs [44]. DC-HIL and PD-L1 expressing MDSCs have displayed more potent suppressive activity, including reducing autoantibody levels, inhibiting double-negative (CD4^−^CD8^−^CD3^+^) T cells and follicular helper T cells, and mitigating podocyte damage [15, 66]. The expressions of DC-HIL and PD-L1, as acquired by MDSCs under autoimmune environments, are examples of how MDSCs possess different efficacies in relation to suppression. However, this trend was broken by the observation that LOX-1^+^ M-MDSCs promoted Th17 differentiation and exacerbated disease development in the inflammatory environment [17]. Thus, the complexity of MDSC phenotypes could break the consistency of MDSC suppression.

Another study with the similar conclusion showed that epigenetic modifications in MDSCs also influenced their function plasticity. In hospitalized sepsis survivors, only MDSCs obtained at and beyond 14 days post-sepsis displayed unique epigenetic (miRNA) expression patterns and notably suppressed T lymphocyte proliferation, compared to earlier time points [99]. Furthermore, the over-expression of long non-coding RNA NEAT1 in PMN-MDSCs was found to promote IFN-I inducible gene expression, CD69 expression, and phosphorylation of JAK1 and STAT1 in IFN-α-stimulated B cells, ending with the disease progressing [23]. The genetic and biological diversity of MDSCs intrinsically shaped their diverse function.

Moreover, the classical phenotype of MDSCs is not specific. Neutrophils and immature PMN-MDSCs are phenotypically and morphologically similar, with a common origin, especially low-density neutrophils (LDNs) [100]. Previous studies have shown that LDNs involved mature neutrophils and immature PMN-MDSCs, and acquired non-cytotoxic, reduced migration, suppressive, and pro-tumor effects in cancer progression [101]. Recently, several recent reports have indicated that LDNs play a pathogenetic role in systemic autoimmunity. Activated LDNs did not inhibit CD4^+^ T cell proliferation in an arginase-dependent manner and stimulated Th1 responses via inducing pro-inflammatory cytokine production (TNF-α, IFN-γ, and lymphotoxin α) [102]. Additionally, LDNs were able to synthesize IFN-γ, induce endothelial dysfunction, undergo spontaneous NETosis, and enhance pro-inflammatory and phagocytic capacities, contributing to lupus pathogenesis and end-organ damage of SLE [103]. In particular, the percentage of LDGs increased obviously in MS or neuromyelitis optica spectrum disorder patients compared with healthy donors, pointing toward their shared pathogenetic mechanisms with SLE [104]. As a result, the role of LDGs is not limited to immune suppression in autoimmunity, and the nonspecific phenotype of MDSCs brings about difficulties in distinguishing them from LDGs.

Furthermore, the diversity of MDSC functions should not be ignored. It has been generally considered that MDSCs have an impaired ability to differentiate into mature DCs, macrophages, and neutrophils. As such, there is the possibility that MDSCs share common capabilities with innate immune cells. Firstly, the released cytokines from MDSCs play a pro-inflammatory and pathogenic role in autoimmunity, including Arg-1, NO, ROS, IFN-γ, and IL-1β. Most reports have described that MDSCs use ROS molecules as part of a major mechanism to inhibit T and B cell responses, even DC maturation and natural killer cell toxicity, but ignored the detriment of ROS itself on the surrounding histiocytes [105]. ROS produced by TLR-7-activated MDSCs was able to induce podocyte injury by activating p-38MAPK and NF-kB signaling [21]. Concerning T cells, co‐culturing with IFN-γ-treated PMN-MDSCs impaired Treg differentiation via ROS, breaking Th17/Treg balance and aggravating disease severity [20]. Similarly, the levels of Arg-1 and IL-1β were positively correlated with the percentage of MDSCs and Th17-mediated autoimmune diseases. Secondly, although they are well-known as immunosuppressive regulators in pathological conditions, MDSCs still retain reduced capabilities in relation to migration, phagocytosis, oxidative burst, and inflammation. In particular, MDSCs provide a shelter for some pathogens during infection. MDSCs have also suppressed T cell responses in active tuberculosis, while concomitantly phagocytosing mycobacteriaas reservoir cells and increasing the risk of tuberculosis disease during early infection [106]. The same held true for viral replication in human immunodeficiency virus type 1 (HIV-1) infections. MDSCs changed their activity and function between augmentation of effective protective antipathogen responses and protection from excessive inflammation during infections [107]. Lastly, previous studies have reported that the effects of MDSCs are dependent on cell–cell contact and cytokine generation. Exosomes are single-membrane vesicles which have the capacity to alter the extracellular environment, and deliver molecules and signals to neighboring cells [108]. Some molecules from MDSCs (Arg-1, PGE2, miR-29a-3p, and miR-93-5p) have been reported to inhibit T cell responses and promote IL-10^+^ Breg cell generation in autoimmunity, consistent with MDSC suppressive activity [35, 36, 76]. However, alterations of inflammation-related miRNA expression (miR-155, miR-223, miR-34b-3p, and miR-210) which are verified in MDSC-derived exosomes [109] seem to participate in Th17 cell proliferation and the aggravation of autoimmune diseases [110]. Unfortunately, there is still a short fall of additional observations relating to MDSC-derived exosomes presence and activity in autoimmune diseases. Therefore, MDSCs should not simply be considered as immunosuppressive regulators.

In addition to the mentioned activities and functions, the immune suppression of MDSCs is not always in a highly efficient manner. Adoptive transfer of MDSCs to adequately control the disease progression has not yet been successful; the fault partly lies with the impaired function of MDSC suppression. Interestingly, on a per-cell basis, the potential of MDSC suppression is also variable under different conditions. M-MDSCs from arthritis animals achieved the strongest suppression levels on T cell proliferation at a 1:4 ratio, while M-MDSCs from control animals did not reach the equivalent suppression at any ratio, but performed poor suppressive capacity at 1:1 and 2:1 ratios [111]. A semblable result was demonstrated in the T1D murine model where MDSC transfer prevented diabetes onset mostly at lower MDSCs doses [93]. On the contrary, a case refuted that the suppression of M-MDSCs on autologous T cells in T1D patients was dependent on cell–cell contact and TGF-β production only at the higher MDSCs:T cells ratio [90]. In conclusion, the evidence indicating the suppressive activity of MDSCs is not stable under different inflammatory conditions and throughout disease progression.

At last, the essential factor to consider is the alteration of the local milieu. Cytokines, immune regulatory molecules, and transcription factors can alter the recruitment, suppressive potency, and survival of MDSCs [112]. For example, tumor glycolysis induces the expression of LAP by inhibiting the AMPK-ULK1-activated autophagy signaling pathway, to efficiently control the expression of G-CSF and GM-CSF, and eventually support MDSC development and maintain tumor immunosuppression [113]. MDSCs after incubation with IL-1β and GM-CSF acquired more efficient suppression than native MDSCs from diseases [89]. Hence, the aggressive inflammation in autoimmunity may gift different manifestation of MDSCs in different diseases. In several autoimmune diseases, MDSCs showed pro-inflammatory effects inducing Th17 cell differentiation as described above. Recently, more studies on SLE as above-mentioned have revealed the pathogenic role of MDSCs in immune responses. Metformin application reduced M-MDSC differentiation via the AMPK/mTOR signal pathway signal and attenuated lupus symptoms in pristane-induced lupus mice [114]. In addition, the infiltration of MDSCs sculpted their effects with tissue specificity. In most cases, MDSCs also preserved immunosuppressive functions including suppression of DC maturation, limiting T cell responses, and controlling B cell accumulation in the inflamed tissues [30, 54]. As to organ-specific effects, infiltrated MDSCs were able to generate several cytokines and soluble factors such as ROS and osteopontin, which participated in the tissue damage and repair [21, 60]. In short, the local environment modifies the characteristics of MDSCs by various mediators and crosstalk with the surrounding cells, which in turn regulates the classical functions of MDSCs.

In summary, increasing evidence about MDSCs in autoimmunity, regardless of their protective or pathogenic role, suggests that MDSCs exhibit diverse and inconsistent functions in immune responses, which is associated with their phenotypic diversity and plasticity in different conditions of autoimmune disease. Therefore, further studies should focus on the mechanistic details behind the diversity of MDSC function within autoimmune diseases, to ensure that the protective effects of MDSC-based therapies in autoimmunity are fully elucidated.

## Data Availability

All data supporting the findings of this study are available within the paper.
